# Neuronal chains as processing units of long scale wave information

**DOI:** 10.1186/1471-2202-14-S1-P256

**Published:** 2013-07-08

**Authors:** Jose A Villacorta-Atienza, Valeri A Makarov

**Affiliations:** 1 Dept. of Applied Mathematics, F. CC. Matemáticas, Universidad Complutense, Madrid 28040, Spain

## 

Information processing in the brain relies on the functional cooperation of individual neurons and neuronal nuclei. It is widely accepted that such cooperation may occur in time domain through synchronous firing of multiple, including spatially distant, neurons. Complementary, the brain, as spatially extended complex structure, may also employ coding and processing of long-scale information in spatial dimension. This alternative, significantly less explored in the literature, recently received experimental support. Here we propose a novel concept of spatiotemporal representation and processing of long-scale information in laminar neural structures. According to this idea, relevant information may be coded in self-sustained traveling waves of neuronal activity whose nonlinear interaction yields efficient wave-processing of spatiotemporal information. We study this concept by modeling the spatially extended neural activity in a chain of FitzHugh-Nagumo neurons with an additional voltage-gated membrane current. This local mechanism turns nonlinear traveling waves in a carrier of long-scale information. Long scale waves can interact between one another, which may lead to complete or asymmetric annihilation and transparent crossing. Then the wave interaction can selectively change information contained in the neuronal structure. We show that laminar neuronal structures exhibit a variety of functionally different regimes including, e.g., decimation of the input stimuli. Moreover they can selectively compress stimuli in the natural frequency range and process them differently by considering the context in which they appear, i.e. other waves involved in the processing. Thus neuronal chains can work as processing units performing different operations over spatiotemporal information.

